# An ecometric analysis of the fossil mammal record of the Turkana Basin

**DOI:** 10.1098/rstb.2015.0232

**Published:** 2016-07-05

**Authors:** Mikael Fortelius, Indrė Žliobaitė, Ferhat Kaya, Faysal Bibi, René Bobe, Louise Leakey, Meave Leakey, David Patterson, Janina Rannikko, Lars Werdelin

**Affiliations:** 1 Department of Geosciences and Geography, University of Helsinki, PO Box 64, Helsinki 00014, Finland; 2 Centre for Ecological and Evolutionary Synthesis, Department of Biosciences, University of Oslo, PO Box 1066 Blindern, Oslo 0316, Norway; 3 Museum für Naturkunde, Leibniz Institute for Evolution and Biodiversity Science, Berlin 10115, Germany; 4 Helsinki Institute for Information Technology HIIT, PO Box 15600, Aalto 00076, Finland; 5 Departamento de Antropología, Universidad de Chile, Santiago, Chile; 6 Turkana Basin Institute, Nairobi, Kenya; 7 Department of Anthropology, Stony Brook University, Stony Brook, NY 11794, USA; 8 Department of Anthropology, Center for the Advanced Study of Hominid Paleobiology, The George Washington University, Washington, DC 20052, USA; 9 Department of Palaeobiology, Swedish Museum of Natural History, PO Box 50007, Stockholm 104 05, Sweden

**Keywords:** fossil mammal, palaeoclimate, ecosystem change, human origins, predictive modelling

## Abstract

Although ecometric methods have been used to analyse fossil mammal faunas and environments of Eurasia and North America, such methods have not yet been applied to the rich fossil mammal record of eastern Africa. Here we report results from analysis of a combined dataset spanning east and west Turkana from Kenya between 7 and 1 million years ago (Ma). We provide temporally and spatially resolved estimates of temperature and precipitation and discuss their relationship to patterns of faunal change, and propose a new hypothesis to explain the lack of a temperature trend. We suggest that the regionally arid Turkana Basin may between 4 and 2 Ma have acted as a ‘species factory’, generating ecological adaptations in advance of the global trend. We show a persistent difference between the eastern and western sides of the Turkana Basin and suggest that the wetlands of the shallow eastern side could have provided additional humidity to the terrestrial ecosystems. Pending further research, a transient episode of faunal change centred at the time of the KBS Member (1.87–1.53 Ma), may be equally plausibly attributed to climate change or to a top-down ecological cascade initiated by the entry of technologically sophisticated humans.

This article is part of the themed issue ‘Major transitions in human evolution’.

## 1. Introduction

The Turkana Basin holds a special place in palaeoanthropology because of its unique record of Plio-Pleistocene hominin evolution. Fieldwork conducted in the basin over decades has generated a highly resolved contextual framework for the hominins, as well as large collections of other fossils, including an exceptionally dense record of mammals [1–14]. These fossil mammals play an important role in the growing understanding of changing environments and climate of the Turkana Basin, and several approaches have been used in their study. These include analyses of diversity, turnover [15,16], community structure in terms of body size, locomotion and diet [17,18], and habitat and diet based on stable isotopes preserved in dental enamel [19–21] and paleosol carbonates [22–26]. Hernández Fernández & Vrba [27] used principal component analysis of fossil mammal faunas to generate the first quantitative estimates of Plio-Pleistocene precipitation in the Turkana Basin. Missing until now have been studies based on dental ecometrics as recently developed in a Eurasian fossil context [28]. Here we present the first results of estimates of climate and environments of the Turkana Basin based on dental ecometrics.

The ecometric approach is used here for the first time in an African context and is methodologically independent of previous work mentioned above [15–27]. It is based on identifying trait—environment relationships and using them to estimate environmental parameters in space and time [29]. The first such ecometric to be explicitly developed, mean-ordinated hypsodonty (molar crown height) of large mammal herbivores, was used to map continental-scale precipitation patterns in the Neogene of Eurasia [30]. Subsequently, a methodology giving numerical estimates of rainfall in mm/yr was developed [31,32]. Combining hypsodonty with another ecometric, occlusal cutting-edge count, improved predictions of global rainfall and also allowed prediction of temperature and primary productivity [33]. For the world today, this combination resolves *ca* 70% of terrestrial net primary productivity, similar to the best available alternatives. Intuitively, the fundamental property being estimated is some aspect of productivity, such as the growth of new, edible plant matter. The less there is, the more demanding will be the task of the teeth in chewing hard, dry foods of poor nutritive quality. Because productivity in most terrestrial environments mainly depends on humidity and temperature, it is not surprising that these climate variables can be estimated directly from dental ecometrics.

The ecometric method could, in principle, be ‘taxon-free’, because it uses morphological information with no regard to taxonomy or taxon identification. In reality, however, ecometrics relies on taxonomy for propagating the relevant information across occurrences. Any fossil of the modern horse tribe Equini, correctly identified, can, e.g., be confidently classified as hypsodont with two longitudinal lophs (cutting edges) on its molar teeth, regardless of whether that morphology is actually present on the fossil in question. In some cases, the ecometric scores of entire families can be set in this way; in others, taxonomic resolution at the genus or even species level is required. It follows that the quality of taxon identification is crucial and also that all sufficiently precisely identified fossil occurrences in space and time can be used, rather than only the few trait-bearing specimens, maximizing sampling density.

It is a property of ecometric analysis that it captures signals at short timescales, despite the fact that the evolution of hypsodonty—as well as deep structural properties such as the number of cutting edges—is typically much slower than environmental change. Ecometric patterns reflect changes in the distribution of taxa as found, not their morphological evolution, and is thus a phenomenon taking place on ‘ecological’ rather than on ‘evolutionary’ timescales in the conventional sense. It follows that ecometric patterning, while at any one time probably reflecting the distribution of selection pressures, does not possess phylogenetic or other inertia, and in that sense is comparable to other short-term signals, such as sedimentary properties and stable isotope ratios.

Ecometric methodology was developed in a conventional structure of ‘localities’ with age, location and a list of occurring taxa. This poses a challenge for applying ecometrics to the fossil record of the Turkana Basin, where each specimen has independent information about its placement in space and time. In this first attempt, we have created computational, locality-like entities called ComLocs, by aggregating specimens in space and time. Spatial aggregation is by 'place', an entity combining collecting area, site and locality, as used in the Turkana database, whereas temporal aggregation is by stratigraphic Member. Such ComLocs are probably, on average, more inclusive than a typical fossil ‘locality’, with greater temporal and spatial extent and averaging, but essentially similar collections of fossil specimens representing a place in time. Moreover, ComLocs should make the direct comparison of modern-day data with fossil occurrences more straightforward. The ComLoc is in any case a finer-scale aggregate than the time bins that have typically been used in analyses of temporal trends in these data and permits resolving the data spatially in map form. For maximum compatibility with earlier work, we also aggregate the data by 0.4 million year time bins and plot the corresponding trend lines.

This is a first attempt and inevitably suffers from shortcomings of data and methodology. We accordingly report our results and interpretations in a tentative and cautious spirit as hypotheses to be verified or rejected by later work.

## 2. Material and methods

### (a) Data

For this study, we complemented the Turkana Basin Paleontology Database^1^, created by a collaborative project between the National Museums of Kenya (NMK) and the Smithsonian Institution, with several datasets for Lothagam, Kanapoi and sites on the western side of Lake Turkana, respectively, from files curated by Meave Leakey. We also added data for both east and west sides collected since 2005 until 2009 inclusive and updated the taxonomic identifications of monkeys (ML), carnivores (LW) and bovids (FB, DP, RB). The combined dataset, limited to the Kenyan part of the Turkana Basin, was pruned to exclude non-mammal records, and any records not yet accessioned by the NMK. The dataset currently comprises 19 927 records, corresponding to 14 581 specimens assigned to 345 ComLocs, out of which 139 ComLocs with 11 748 specimens qualified for analysis. Among these, 2128 unique ComLoc-species were identified and used for producing precipitation and temperature estimates. The specimen-level data used along with the resulting temperature and precipitation estimates are available in the electronic supplementary material.

The dataset used for the turnover analysis includes two components. The data on non-carnivorans are the same as those used by reference [34]. The data for carnivorans have been updated by LW for the present analysis to include all unpublished west Turkana carnivorans in addition to the recent analysis of east Turkana [14].

For temporal resolution, we used all available information at the level of stratigraphic Member or finer [35]. For spatial coordinates, we used the midpoints of the collecting areas, sites and localities (‘places’) listed in the database. Computational localities (ComLocs) were created as unique combinations of places and Members. The taxon list for each ComLoc was created by listing all the unique taxa represented and pruning it under the conservative assumption that no taxa recorded at a higher level represented taxa unrecorded at a lower one (e.g. if *Menelikia* indet. was recorded, it was assumed that no other taxon was present among tribe Reduncini from the same ComLoc, unless explicitly recorded at the genus or species level).

Ecometric information (hypsodonty and lophedness) was added to the dataset by MF based on a combination of collections work and expert knowledge. Hypsodonty (HYP) was scored as in reference [31]. Scores were based on observations at the species or genus level, except for family Bovidae, where a conservative scheme was applied at the tribe level, such that all bovids were scored as hypsodont (3), except for tribes Tragelaphini and Boselaphini, which were scored mesodont (2). Simple experiments suggested that the choice between this and other plausible but more complicated alternatives had little effect on the mean values obtained. Longitudinal lophedness (LOP) was scored as 0, 1 or 2, according to criteria in reference [36]; transverse lophs were not scored [33].

Data from the NOW database [37] were downloaded in August 2015 and used for the comparison of Turkana Basin ecometrics with eastern Africa. This dataset was harmonized for HYP and LOP scores with the Turkana Basin data.

Predictive models for estimating temperature and precipitation were inferred from modern species occurrence data. The methodology of Liu *et al.* [33] was used, although the underlying base models were different, and instead of WWF ecoregions, we used International Union for Conservation of Nature modern occurrence maps^2^. Processed modern-day data were obtained from reference [38]. Climate layers from the WorldClim (worldclim.org) database [39] were obtained from reference [38]. The original data were gridded by latitude and longitude to 50 km resolution. The WorldClim data include nineteen bioclimate variables [40], from which we selected two for modelling: mean annual temperature (bio1) and annual precipitation (bio12). These are expected to capture principal climatic patterns and trends. Although we acknowledge that climatic averages are rarely biologically limiting, we felt they would be the most intuitive and generally understandable metrics to use for this pilot study.

### (b) Methods

#### (i) Methods for carnivore analysis

The turnover analysis used the methodology introduced by Foote [41]. Carnivorans and non-carnivorans were analysed separately to investigate possible differences in turnover between trophic levels. Data were binned into 300 kyr bins (the smallest the data would support). The binning process was the same for both carnivorans and non-carnivorans.

#### (ii) Models for estimating climate variables

Following the methodology of Liu *et al*. [33], we built regression models for separately estimating temperature and precipitation from mean HYP and mean LOP. Modern-day data were divided into grid cells (to mimic computational localities of the fossil record), each grid cell had an associated species occurrence list and climate parameters. For each grid cell, average dental trait values were computed taking into account occurring species. These average trait values were used as inputs for predictive models with the climate parameters as target variables.

In earlier studies, models were fitted on global data [31,33]. We have observed that modern-day distributions of traits substantially differ across continents owing to palaeobiogeographic effects. In this study, we therefore fit climate models on African data within 25 degrees of the Equator, expecting these data to be the closest capture of trait distribution of the Turkana region in the past, and at the same time broad enough to incorporate a range of possible climatic conditions. The model coefficients were estimated on a subset of observations that had at least three species. After filtering, we had 7479 observations for fitting the model. Precipitation and temperature required different base models. Because the signal for precipitation is much stronger than for temperature in the HYP and LOP data, a nonlinear model is chosen for precipitation, whereas a more conservative linear model is chosen for temperature.

To estimate precipitation, we used a nonlinear regression model with an interaction term. This model was selected from several alternatives by visual inspection of relations between HYP, LOP and precipitation in the data. Additional verification of model form selection was done via cross-validation. Ordinary least-squares procedure was used for estimating the model coefficients. The resulting model is2.1
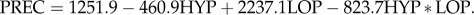


To estimate temperature, we used a linear regression model. Model coefficients were estimated using the principal component regression procedure (implementation from pls package in R^3^) with one component. As LOP is strongly linearly correlated with HYP, and at the same time, the relation between HYP, LOP and temperature is weak, we were seeking a conservative model that would produce robust estimates without too extreme deviations from the mean temperature, hence, principal component regression suited the purpose. The model is fitted by first making a linear projection of data such that the new variables are minimally correlated with each other and maximally correlated with the target variable (temperature). A regression model is then fitted to the projected variables. Even though obtained differently, the resulting model looks and behaves like a standard linear regression. The fit is weaker than for precipitation, but we believe it is still sufficiently robust to capture generic underlying trends. The resulting model is2.2
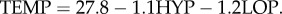


Apart from regression models, which are commonly used in paleoecology, we experimented with non-parametric k-nearest neighbours (kNN) models, which allow modelling complex patterns without assuming the form of relation between variables. kNN allows closer fit to the data, but is more difficult to interpret. While a regression model is a formula, kNN model is a collection of examples. The kNN closely follows data; for example, it cannot produce negative precipitation if there is none in the data, whereas a regression model may produce negative precipitation. kNN does not perform explicit generalization, but predicts by comparing a new example with the *k* closest reference examples, stored in memory. We used *k* = 15, which was selected from a range of options via cross-validation on the modern-times data.

**Figure 1.**
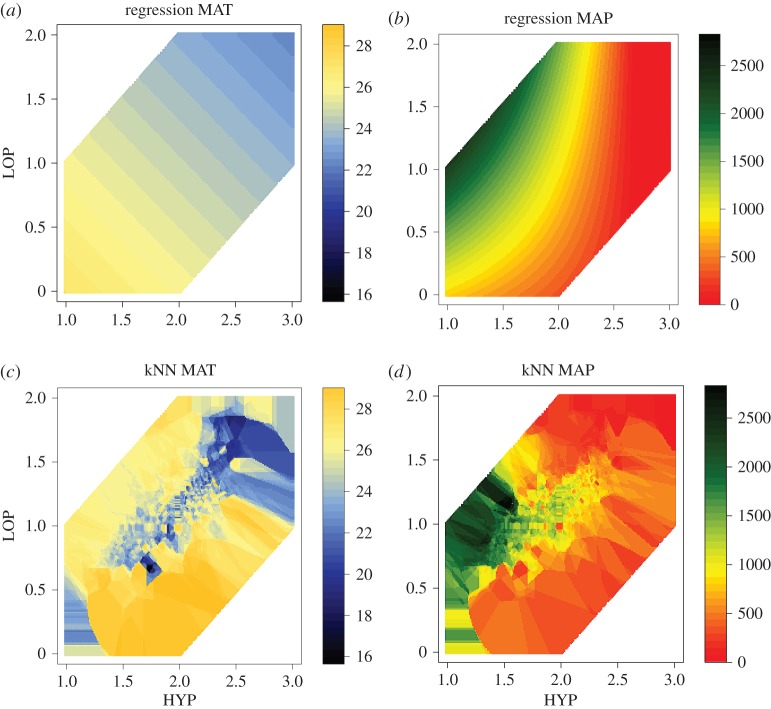
Visualization of the models for temperature and precipitation. Interpretation for all figures is as follows: given a ComLoc in question one measures mean HYP, mean LOP and then finds a point on the plot corresponding to the obtained measures. The colour-coding at this point gives an estimate for temperature or precipitation.

Figure 1 visualizes the resulting models for temperature and precipitation. This is a visualization of the model decision space, rather than actual observations. This is to demonstrate how the models behave at different values of HYP and LOP. We can see that the regression models make a uniformly gradual transition from warm to cold, and from wet to dry, whereas kNN models, for instance, capture wet spots more abruptly. The two models present two extreme ends of generalization versus closely following data—the reality is perhaps somewhere in between. We believe that both models (regression and kNN) have their merits. Therefore, our analysis presents and discusses results obtained using both.

#### (iii) Methods for fossil data analysis

Ecometric temperature and precipitation estimates obtained by regression and kNN models for time intervals corresponding to aggregates of fossil taxa by 0.4 Ma time bin (a binning that best accommodates the Member age ranges), by Turkana Lake Phase, by Member and by ComLoc, were analysed using standard statistical and visualization techniques. Because the regression model may occasionally produce a negative estimate for precipitation, we post-processed all such estimates to 0.

We analysed the data in two modes: aggregated primarily in space and aggregated primarily in time. Even though the gaps between sampled time points are not even over time and some interim details are lacking, generic trends can be clearly observed. Thus, the oldest records (Lower and Upper Nawata) appear as single ComLocs per time unit because they cannot currently be further divided temporally or spatially. In the plots, these averages stand as single points, but volume-wise, the estimates are comparable to averaging over several points elsewhere, which themselves come from smaller assemblages. We also undertook a detailed study of the fossil data from the temporal and spatial setting of the ‘KBS event’ that we recognized. For aggregation in time, we used local polynomial regression (implementation in R stats package^3^), with degree of smoothing 0.75, degree of polynomial 1 and fit by least-squares. The 95% CI of the fit is indicated in the figures. For aggregation in space, we used inverse distance-weighted interpolation in QGIS^4^, with settings adjusted case by case (see figure captions). The values of individual localities and ComLocs are shown as highlighted circles on a faded interpolated background [30].

## 3. Results

### (a) Ecometric precipitation and temperature estimates for the Plio-Pleistocene of Turkana

**Figure 2.**
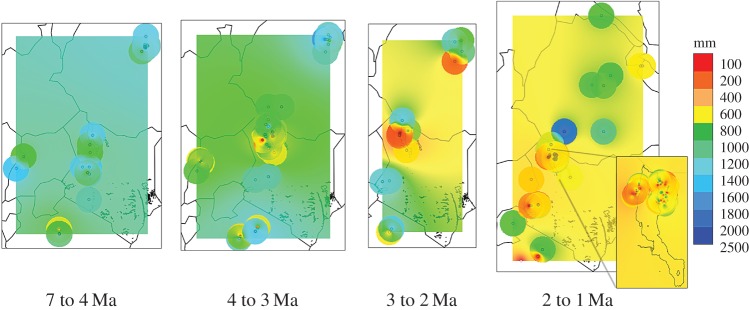
Regional distribution of ecometric estimates of mean annual precipitation (mm/yr) in eastern Africa during four intervals. Inset map shows distribution within the Turkana Basin for the last interval. GIS settings: inverse distance weighting with distance coefficient 2.0, radius 1: 1.0, radius 2: 1.0, resize width: 3000, resize height: 3000.

Within the regional setting of eastern Africa, operationally defined here as the territories of present-day Ethiopia, Kenya, Uganda and Tanzania, the Turkana Basin appears relatively arid during much of the interval studied (figure 2). It has lower precipitation estimates than the surrounding areas overall. This difference is mostly owing to the interval 4–2 Ma (*t*-test, 95% confidence of means of regression model estimates, *p* < 0.0001), with regional aridity especially marked (with about half the rainfall of surrounding areas) during the Moiti Floodplain (4.0–3.6 Ma) and Tulu Bor (3.4–2.0 Ma) phases. This contrast is entirely absent during the Lorenyang Lake Phase (2.0–1.4 Ma), when conditions actually appear locally slightly less arid than in the surrounding areas. The Turkana Basin does not appear to differ significantly in aridity from the rest of eastern Africa prior to 4 or after 2 Ma.

**Figure 3.**
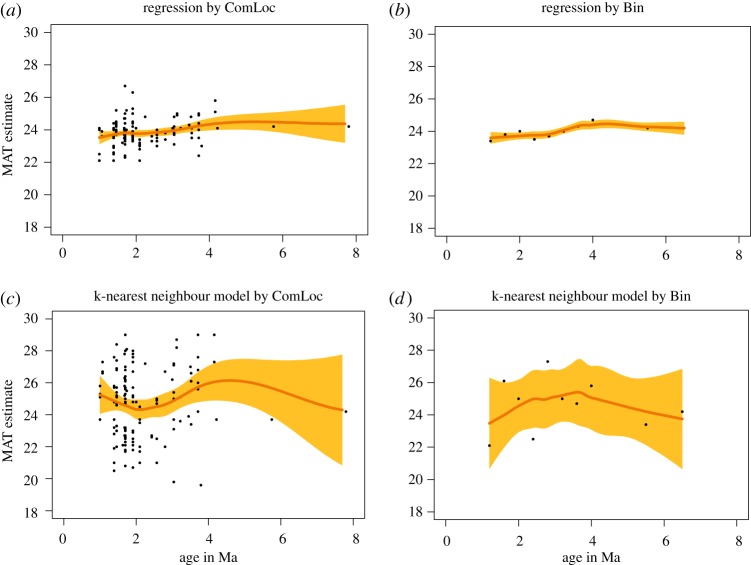
(*a*–*d*) Estimates of mean annual temperature (°C) over time in the Turkana Basin using two different levels of aggregation (right–left) and two different models based on the dental ecometrics HYP and LOP (up–down). 95% CI for the fit are shown in orange; please note that a narrow interval does not imply confidence in the estimated value itself, only in the consistent performance of the model.

According to ecometric estimates, Turkana Basin temperatures remained remarkably constant during the 6 Ma interval studied (figure 3). Whether the mean annual temperature really was *ca* 24°C is moot, but the lack of a long-term trend matches previous results based on soil carbonate isotopes [42]. Depending on the model, there may or may not be a slight indication of a temperature peak near 4 Ma, which might reflect the globally warm early Pliocene [43].

**Figure 4.**
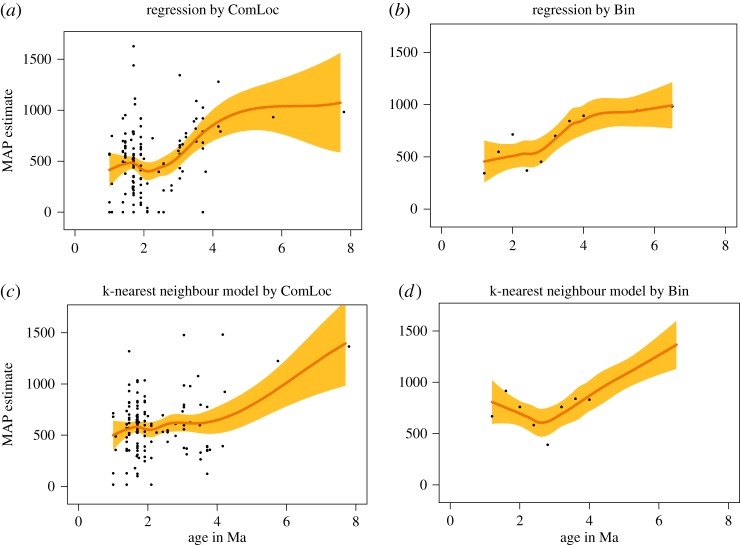
Estimates of mean annual precipitation (mm/yr) over time in the Turkana basin using two different levels of aggregation (right–left) and two different models based on the dental ecometrics HYP and LOP (up–down). 95% CIs for the fit are shown in orange.

As expected from previous studies, humidity in the Turkana Basin declined from the late Miocene, reaching a lower plateau during the Plio-Pleistocene (figure 4). Depending on the model used, the decline was gradual during the Pliocene or quite abrupt soon after the still-humid time around 4 Ma. In addition, there may be one or two intervals of increased variance. All models show a great spread of values near 1.8 Ma, but only some also show this near 3.5 Ma. It is presently not possible to tell from data or analytical results whether this increased variability indicates increased sampling density, increased spatial heterogeneity or increased temporal variation. However, the fact that such periods of increased variance are not seen for the temperature estimates suggests that something beyond sampling is involved.

**Figure 5.**
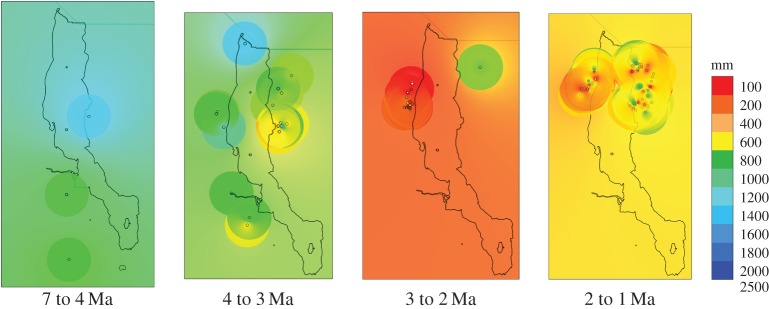
Distribution of ecometric mean annual precipitation (regression model) from ComLocs in the Turkana Basin during four time intervals. The apparent precipitation minimum in the 3–2 Ma interval is partly owing to extremely low estimates from the Kalochoro Member of West Turkana. GIS settings: inverse distance weighting with distance coefficient 2.0, radius 1: 0.2, radius 2: 0.2, resize width: 1000, resize height: 1000.

Ecometric precipitation maps for Turkana show the spatial details of the temporal trends (figure 5). The emergence of an east-west contrast is seen from 4 Ma onwards, as soon as the present tectonic structure was created [44]. The shallow eastern side of the half-graben appears more humid than the elevated western side and, especially after 2 Ma, more spatially heterogeneous, in agreement with previous work [11,45]. The hypothesis that temporal variability also increased regionally as a result of orbital forcing [46,47] is entirely consistent with our results but cannot be independently supported, owing to the low temporal resolution available to us.

**Figure 6.**
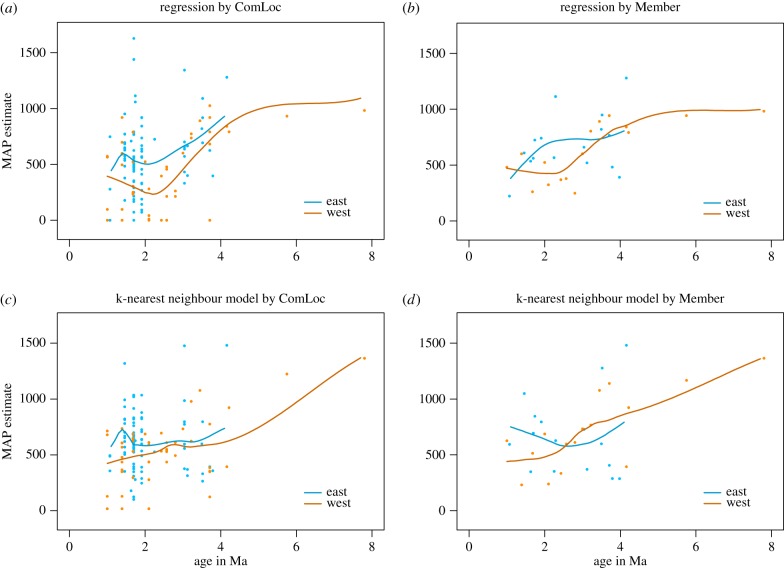
Comparison of mean annual precipitation (mm/yr) between localities from the east and west sides of present-day lake Turkana over time, with the same models and fit as in figures 3 and 4.

Comparing the temporal trends of the two sides of the basin reveals a clear difference over time (figure 6), regardless of resolution (mode of aggregation). The wetter east side has a greater spread of values, possibly reflecting better sampling but perhaps also suggesting greater spatial heterogeneity or greater sensitivity to fluctuations in the water table, related to influx of river water rather than rainfall. Under such an interpretation, the elevation of the estimates from the east side would be due to local surface or ground water and, potentially, teleconnection to climatic changes elsewhere, rather than to greater local rainfall on the east side. This interpretation appears climatologically more plausible and is supported by some of the most humid ComLocs being situated in areas where sedimentology records the presence of river mouths and palaeodeltas [44,48].

**Figure 7.**
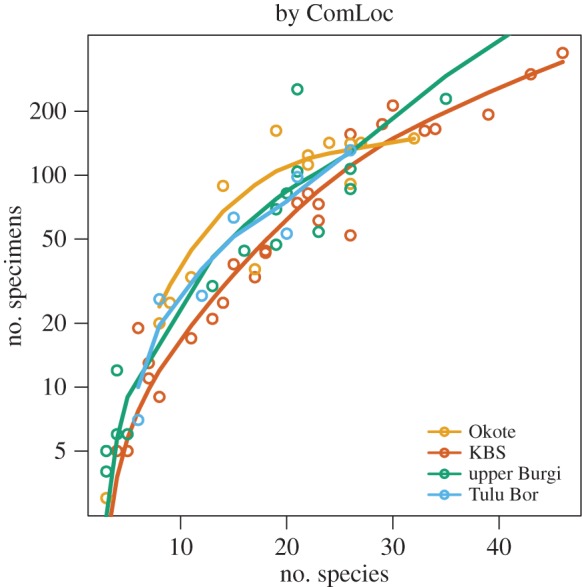
Relationship of species detected to specimens collected in the analysed dataset, showing exceptionally high species/specimen ratio of the KBS Member. The fit is by cubic smoothing spline with 4 degrees of freedom.

The ratio of specimens collected to taxa identified is quite stable in the data but one anomaly stands out: the extremely well-sampled KBS Member at Koobi Fora (1.87–1.53 Ma) has a much lower ratio of specimens to species than do other intervals (figure 7). This includes the preceding and equally well-sampled Upper Burgi Member (2.00–1.87 Ma), suggesting that sampling intensity alone is not the cause. This anomaly corresponds to a major turnover event described below but apart from the possible increase in temporal or spatial variability, there is no indication of a corresponding major local climate effect in the ecometrics (figure 6).

**Figure 8.**
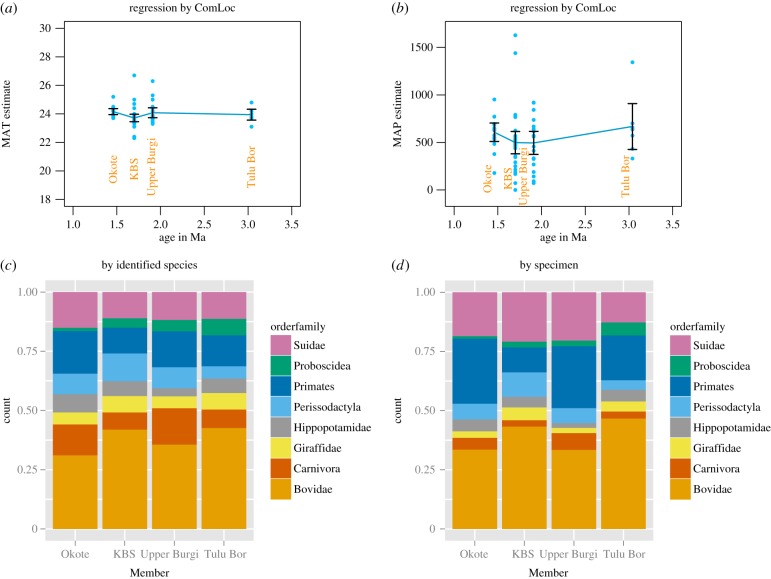
Taxonomic distribution of individual specimens collected from the east-side Members before, during and after the ‘KBS event’. (*a*) Mean annual temperature, (*b*) mean annual precipitation, (*c*) relative proportions of species across taxa, and (*d*) relative proportions of specimens across taxa. Note transient minimum of carnivore and primate specimens during KBS time and corresponding maximum of bovid and perissodactyl specimens.

A key to the nature of this signal is given by detailed comparison of the sequence of Members of the Koobi Fora Formation during this interval: the Upper Burgi, the KBS and the Okote (1.53–1.38 Ma). This comparison shows that (i) the low specimen-to-species ratio is restricted to the KBS Member (figure 7), (ii) as far as can be told, it affects all taxonomic groups, (iii) the KBS Member has a lower temperature estimate, owing to a higher mean LOP value, in turn caused by an exceptionally high number of bovid and perissodactyl species (figure 8*c*). The succeeding Okote Member is, interestingly, an exception in the opposite direction, with an unusually high ratio of specimens to species (figure 7). For what its worth, the ecometric signal of lowered temperature and precipitation suggests slightly decreased, rather than increased, productivity during KBS time.

Figure 8*c–d* also shows that the KBS is characterized by an exceptionally low number of carnivore and primate specimens and an exceptionally high number of bovid and perissodactyl specimens. The two Members bracketing the KBS in time are both quite similar in this regard, despite the fact that the Okote stands out by its higher specimen/species ratio and its higher ecometric estimate of productivity, possibly suggesting a biotic rather than climatic driver of the KBS event.

### (b) Turnover

**Figure 9.**
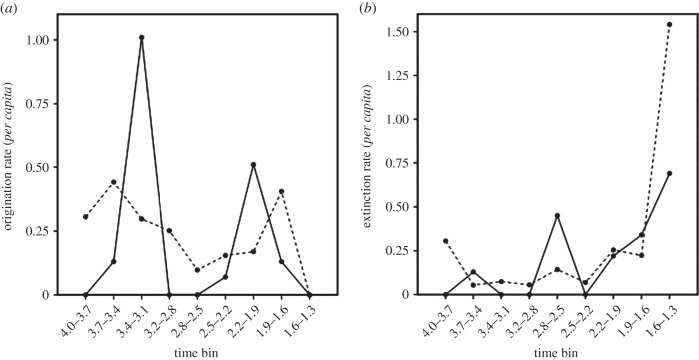
Comparison of turnover patterns in carnivorans and non-carnivorans in the Turkana basin. (*a*) *Per capita* origination showing earlier origination peak in carnivorans (2.2–1.9 Ma) than in non-carnivorans (1.9–1.6 Ma). Carnivoran origination peak at 3.4–3.1 is an artefact. (*b*): *per capita* extinctions showing carnivoran extinction peak at 2.8–2.5 Ma unmatched in non-carnivorans. Taking sampling into consideration suggests that this peak should be at 2.5–2.2 Ma (see Discussion for details). Solid line: carnivorans; dashed line: non-carnivorans.

The results of the turnover analysis are shown in figure 9. Origination patterns are broadly similar in both sets of taxa, with the important exception that the early Pleistocene origination peak occurs one bin earlier in carnivorans than in non-carnivorans. Both datasets concur that origination is at a minimum in the late Pliocene (i.e. 2.8–2.5 Ma). The early Pliocene origination peaks are an edge effect and the difference in timing between carnivorans and non-carnivorans there owing to sampling.

Extinction patterns are more similar between the two datasets, although carnivorans show greater fluctuations; our interpretations will be discussed in §4.

## 4. Discussion

In the regional context of eastern Africa, the Turkana Basin stands out as a relatively arid place for much of the time between 4 and 2 Ma. This pattern potentially matches the concept and setting of a ‘species factory’ [28,49]. While this suggestion may appear to be at variance with previous interpretations of the setting of the Turkana Basin as a refugium, with a more reliable and climate-independent water supply than other basins in the region [27,50,51], it is conceivable that both interpretations may apply. The refugium situation [51] was specifically Lorenyang Lake, when the Turkana Basin, or at least its eastern side, does appear marginally more humid than surrounding areas, whereas the ‘species factory’ scenario would apply particularly to the situation represented by the regionally arid conditions of Tulu Bor and Moiti Floodplains.

Within the Turkana Basin, we find a pattern that has been observed by others [11,13,27]: the east side of the Turkana Basin persistently appears more humid than the west side, and also more variable. Such a pattern might simply be a result of the asymmetric structure and hydrology of the half-graben that has made up the basin the last 4 million years [44]. In this setting, the west side is more elevated above the water table and thus expected to be less influenced by surface water, whereas the low-lying east side will feature local wetlands and therefore show both greater influence of surface water and greater spatial variability. Local changes in rainfall should affect both sides equally, but changes in rainfall outside the sampled area (e.g. the Ethiopian highlands) may impact the east side more.

The ecometric analysis suggests two main conclusions regarding temporal patterns. First, humidity declined from the late Miocene and flattened out at a lower level during the Plio-Pleistocene, in agreement with both marine and terrestrial records that suggest an overall increase in aridity and proportion of C_4_ plant material [23,26,52–55] within eastern African ecosystems during this period. Depending on the model used, our analyses support these findings and suggest a gradual decline in humidity during the Pliocene or an abrupt decline soon after the still humid period around 4 Ma.

Second, temperatures remained remarkably constant during the 6 Ma interval studied. There may have been a slight temperature peak near 4 Ma, consistent with widespread model and proxy evidence for a warm early Pliocene [43]. As for the puzzling lack of overall cooling, we here propose a novel hypothesis. It is well known that deforestation can lead to increased surface temperatures under tropical conditions [56,57], and recent modelling work suggests that the effect is of the same magnitude as the effect of CO_2_-driven global warming today [58]. For central Africa, that study found that 10–20% loss of present-day tree cover would lead to a more than 2°C increase in local temperature, whereas the combined effect of doubled CO_2_ and deforestation would increase local temperature by 3–4°C [58] (figure 5). Analysis of woody cover from hominin sites in the Awash and Omo-Turkana basins suggests an overall trend from predominantly woodland/brushland/shrubland to wooded grassland over the past 6 Ma, corresponding to a decrease in the fraction of woody cover from about 50% to about 30% (figure 6 and electronic supplementary material, figure 1 in ref. [59]). Given a global cooling of about 3°C in the Pliocene [60], a local warming of about the same amount as a result of vegetation changes thus appears entirely plausible for eastern Africa and could account for the lack of a cooling trend in the proxy data.

Overall, we find a plausible match to tectonic history as summarized by Feibel [44]. Prior to 4 Ma, the Turkana region appears regionally relatively humid and internally undifferentiated. With the tectonic reorganization of the early Pliocene the basin becomes a relatively arid region within a more humid context and develops the characteristic differentiation between a dry west and a humid east that remains in place from this time onwards. This internal differentiation remains even after 2 Ma, when Turkana no longer appears more arid than the general background (figure 4). Climatic trends can be discerned on this tectonic template, but it seems to be the eastern side of the graben, with its availability of extra surface water from a large catchment area, that is more sensitive to climatic fluctuations.

There are some indications of climatic phases within the general trend, including increased data scatter after 2 Ma, which in addition to greater spread around the temporal trendline, is also seen as greater spatial heterogeneity on the maps, as previously reported [27]. The increasing temporal variability of global and regional climate around this time is also well known [46,60,61] and is likely to explain some of the observed spread of values.

Locally, the low specimen-to-species ratio uniquely found in the KBS Member (1.87–1.53 Ma) may well be the key to understanding some the processes involved. Specifically, the lack of ecometric support for environmental amelioration (such as increased humidity or primary productivity) leads us to favour non-climatic explanations for the high species diversity, whereas the fact that the phenomenon was only transient argues against explanations based on permanently altered climate or habitats.

It may be no coincidence that sedimentation rates also increased significantly from Upper Burgi to KBS time [24]. All else being equal, increased sedimentation and fossilization would be expected to result in better representation in the fossil record of temporal variability of environments and biota. If species’ ranges shifted with changing climate, this could in turn result in the apparent sympatry of species that were in fact rarely found together in life, inflating the apparent species diversity. Quinn *et al.* [24] attribute the increased sedimentation to the simultaneous spread of grasslands. Needless to say, the spread of open habitats is usually attributed to climate change and could be taken as an indication of local climate change that is not detected by our ecometric proxies. But since nothing suggests that the grasslands subsequently retreated, it is the process of expansion itself, rather than the permanent establishment of a new habitat, which could potentially explain the transiently high richness observed.

It is difficult to draw detailed conclusions from comparisons between turnover patterns of different trophic levels, not least owing to the much smaller samples available for higher trophic levels than lower ones. Nevertheless, there are indications of a decoupling, with the higher trophic level, i.e. carnivorans (strongly dominated by hypercarnivores [14,62,63]) having a somewhat earlier origination peak and markedly earlier extinction peak. That said, however, the time bin 2.5–2.2 Ma is notably poorly sampled for carnivorans, with only a handful of specimens available from west Turkana and none from east. If this time bin is ignored, in the sense that all taxa are allowed to extend through this bin in either direction, the origination peak is moved one bin earlier and the extinction peak one bin later, putting both in the 2.5–2.2 Ma time bin. An intermediate pattern is more likely, but in either case, manipulating the data in this way does not synchronize carnivorans with non-carnivorans.

In the absence of ecometric evidence for distinct climatic changes affecting the local ecosystem to explain either the pattern of differences between trophic levels or each trophic level by itself, it is tempting to associate them with biotic interactions, in particular with a top-down cascade initiated by the entry of technologically advanced humans and collapse of the large carnivore community. Such a cascade could, in principle, explain not only the trophic details of the turnover sequence, but also the spread of grasslands through expanding herbivore populations, overgrazing of woody vegetation and resultant loss of woody cover. More detailed work remains to be done before such a conclusion could be considered justified, however. Regardless of whether the primary cause was climatic, anthropogenic or something else, a close causal link is likely to exist between local vegetation change and the episode of faunal change observed.

## 5. Conclusion

Ecometrics appears able to resolve not only regional differences in climate within eastern Africa, but also differences in conditions within the Turkana Basin. Whereas precipitation estimates show the expected decrease from over 1000 to less than 500 mm/yr over the last 6 Ma, temperature estimates are remarkably stable at about 24°C, possibly reflecting a balance between global cooling and local heating from progressive deforestation resulting from an increased prevalence of grassland-dominated biomes. Exactly how our estimates compare with the significantly higher estimates of soil temperatures based on the clumped isotope analysis of fossil soil carbonates [42] cannot be resolved at present, but the lack of a temporal trend is similar. We strongly encourage workers in other fields to further test this hypothesis. Whether the absolute values are realistic remains to be assessed, but the trends match previously published results. The presence of water on the surface appears to inflate local rainfall estimates significantly, as expected when wetlands supply water to local ecosystems. The more humid estimates obtained for the eastern side of the basin are interpreted as an effect of wetlands on the shallow, eastern slope of the half-graben.

The richness and turnover anomaly observed in the KBS Member is found to correspond to an exceptionally high ratio of the number of species to the number of specimens. As the ecometric analysis does not indicate local effects of climate change as a likely cause, the possibility of a human-induced ecological cascade to explain this and the turnover patterns observed should be further investigated. We observe a substantial but transient decline in the relative number of carnivore specimens from Upper Burgi to KBS, with a partial return towards the preceding state in Okote, an observation not incompatible with a hypothesis of human interference. One possible avenue to further test the hypothesis of human interference would be a detailed study of the trophic structure of the communities, including analysis of body size distributions before, during and after the event.

Several authors [27,50,51] have suggested that the Turkana Basin behaves as a refugium because of the constant water supply of the Omo River, independently of local climate. Our results suggest that this situation does apply part of the time, including the interval targeted by Joordens *et al.* [51], but that the Turkana Basin was also more arid than the rest of eastern Africa for much of the Plio-Pleistocene, especially during the interval 4–2 Ma. This makes the Turkana Basin a candidate for the ‘species factory’ phenomenon in the sense of Fortelius *et al.* [28,49], a situation where local adaptation causes newly arisen species to be pre-adapted to the conditions that will be increasingly widespread in subsequent time intervals. Under such an interpretation, it would be no surprise if new biodiversity was generated there, including new hominin species, ahead of the global drying trend, but buffered from local climate change by river-fed wetlands.

## Supplementary Material

Data supplement

## Figures and Tables

**Figure 10.**
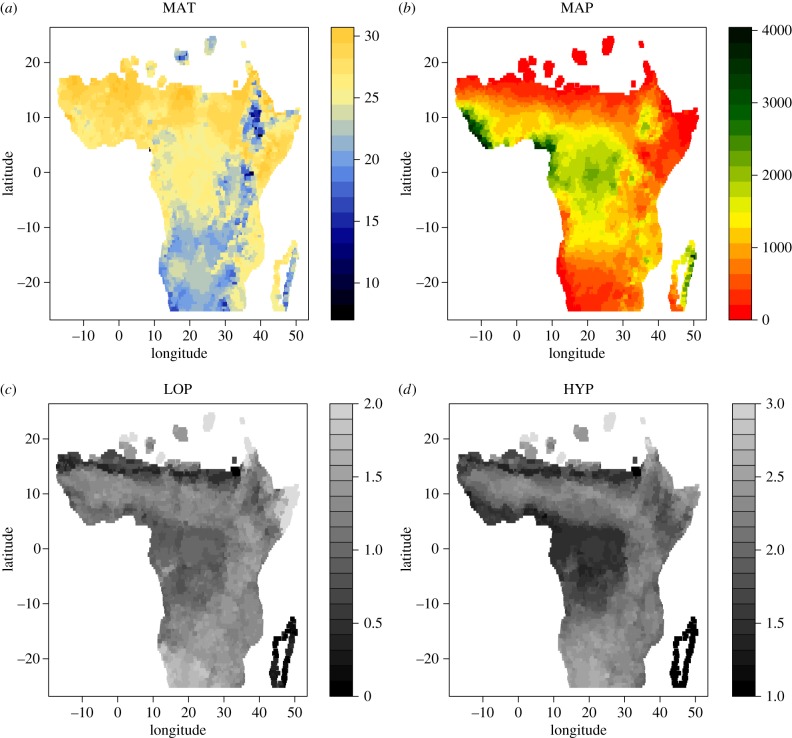
(*a*–*d*) Visualization of the modern-day data used for modelling.

## Endnotes

1 http://naturalhistory.si.edu/ete/ETE_Datasets_Turkana.html.

2 http://www.iucnredlist.org/technical-documents/spatial-data.

3 https://www.r-project.org/.

4 http://www.qgis.org/en/site/.

## Data accessibility

The datasets supporting this article have been uploaded as part of the electronic supplementary material.

## Appendix A

**(a) Additional description of the modelling procedure**

Figure 10 visualizes our modelling data from modern-day Africa within 25 degrees of the Equator. To minimize potential noise in the signal our modelling data excludes grid cells with less than three species, and cells with missing values for LOP (rare Primates; concern less than 0.5% of the grid cells). The data that are visualized in figure 10 have been used for inferring models.

Modelling considers grid cells as observations, average HYP and LOP for each grid cell as model inputs, and temperature or precipitation (separately) as model outputs. We chose different model forms for temperature and precipitation, because the strength of relationship in the modern-day data was different. For precipitation, we used a second-order regression model in order to capture a nonlinear pattern, manifesting as a highly wet spot at around HYP = 1 and LOP = 1 (as can be seen, for instance, from kNN MAT model in figure 1). We have selected this model from several linear and nonlinear alternatives, including variants of subsampling of the modelling data. The selection criteria were a combination of qualitative visual inspection of the data and the resulting decision space (figure 1), and quantitative measures, such as *R*^2^, obtained on the modelling data and via hold-out tests (cross-validation). The fit of the regression model is moderate (*R*^2^ = 0.36), but we believe it provides a good balance between capturing general patterns and following the modelling data. In case the reader is interested in the significance of the regression coefficients, they are all deemed significant (*p* < 0.001), because our modelling sample is large (7479 observations).

Earlier models for temperature [33] were able to capture temperature somewhat well on the global scale, distinguishing very cold negative temperatures from very warm, but were quite volatile on a more fine-grained scale in Africa. The challenge with modelling temperature in Africa is that the relation with HYP and LOP is very weak here, as can be seen, for instance, from figure 10, where low HYP and LOP values are mainly wrapped in the central part, whereas the temperature gradient generally goes from north to south. Therefore, the fit of the model is weak (*R*^2^ = 0.06); however, visual inspection of figure 1 suggests that it still captures the main trend. Our intuition suggests that the relation with dental traits here is weak, because temperature is generally not the limiting factor for food availability in Africa. Therefore, we were seeking a robust model that would capture nothing but the basic relation. Thus, we used a linear regression model fitted with the partial least-squares procedure, which projects the input and the target variable to a new space to explain maximum variance in the inputs. This procedure is particularly well suited for handling correlated inputs. For the final regression model, we use one component, which means the very first level of projection. In other words, we want the model to be robust and stable, to capture only the principal trends in the data and as little noise as possible. This model would tend to conservatively predict values close to the mean.

The next paragraphs discuss some insights into the resulting models; the discussion is based on visualizations of the decision space in figure 1. We can see nonlinear patterns emerging as an ellipse in the diagonal centre of the plot. The surrounding areas are monochromatic mainly, because we have no reference examples far from the diagonal centre, in parts of ecometric space that are sparsely populated if at all. We see medium temperatures in the diagonal centre, with colder spots at both ends (high HYP and LOP, as well as low HYP and LOP). The hottest spots (intense yellow) are at low LOP and medium-to-low HYP, and they are very near a cold spot (dark blue). Whether this pattern is true, in reality, requires further investigation, but it is present in the modelling data, and kNN can capture it, while linear regression cannot capture such patterns, because in linear regression the transition from cold to hot is forced to be smooth and is restricted to linear. That can be seen from figure 1, which presents our regression models for temperature and precipitation, when compared with kNN models.

Similar to temperature, the kNN model for precipitation captures several distinct regions of wetness and dryness nearby, as seen from figure 1. We see medium precipitation on the diagonal, and very dry and very wet spots just above the diagonal. The nonlinear regression model also captures the wet (dark green spot). The main observed difference between kNN and regression models is that the wet sector in the regression is much wider than in kNN. Thus, regression is not able to capture the dry spot at mid-HYP and high-LOP that well, but it provides a functional approximation (an equation), which is more straightforward to reuse than kNN.

**(b) Data and code**

The processed data containing the main results are made available as an electronic supplementary material to this paper. The tab-delimited text file Fortelius_et_al_data.csv contains the processed data used for this paper and the estimates of mean annual temperature and mean annual precipitation by ComLocs obtained.

In addition, for those interested in hands-on computational experiments and plots, we have made available on GitHub^5^ the code in R for fitting the models on modern data, and producing result plots on the fossil data.
